# Establishment of Highly Transplantable Cholangiocarcinoma Cell Lines from a Patient-Derived Xenograft Mouse Model

**DOI:** 10.3390/cells8050496

**Published:** 2019-05-23

**Authors:** Kulthida Vaeteewoottacharn, Chawalit Pairojkul, Ryusho Kariya, Kanha Muisuk, Kanokwan Imtawil, Yaovalux Chamgramol, Vajarabhongsa Bhudhisawasdi, Narong Khuntikeo, Ake Pugkhem, O-Tur Saeseow, Atit Silsirivanit, Chaisiri Wongkham, Sopit Wongkham, Seiji Okada

**Affiliations:** 1 Division of Hematopoiesis, Joint Research Center for Human Retrovirus Infection and Graduate School of Medical Sciences, Kumamoto University, Kumamoto 860-0811, Japan; kulthidava@kku.ac.th (K.V.); ryushokariya@gmail.com (R.K.); 2 Department of Biochemistry, Khon Kaen University, Khon Kaen 40002, Thailand; kanoim@kku.ac.th (K.I.); atitsil@kku.ac.th (A.S.); chaisiri@kku.ac.th (C.W.); sopit@kku.ac.th (S.W.); 3 Cholangiocarcinoma Research Institute, Khon Kaen University, Khon Kaen 40002, Thailand; chawalit-pjk2011@hotmail.com (C.P.); cyaova@kku.ac.th (Y.C.); joevajara@gmail.com (V.B.); knaron@kku.ac.th (N.K.); akepug@kku.ac.th (A.P.); otusae@kku.ac.th (O.-T.S.); 4 Department of Pathology, Khon Kaen University, Khon Kaen 40002, Thailand; 5 Department of Forensic Sciences, Khon Kaen University, Khon Kaen 40002, Thailand; mkanha@kku.ac.th; 6 Department of Surgery, Faculty of Medicine, Khon Kaen University, Khon Kaen 40002, Thailand

**Keywords:** cholangiocarcinoma, patient-derived xenograft, cell line, cancer model, precision medicine

## Abstract

Cholangiocarcinoma (CCA) is a deadly malignant tumor of the liver. It is a significant health problem in Thailand. The critical obstacles of CCA diagnosis and treatment are the high heterogeneity of disease and considerable resistance to treatment. Recent multi-omics studies revealed the promising targets for CCA treatment; however, limited models for drug discovery are available. This study aimed to develop a patient-derived xenograft (PDX) model as well as PDX-derived cell lines of CCA for future drug screening. From a total of 16 CCA frozen tissues, 75% (eight intrahepatic and four extrahepatic subtypes) were successfully grown and subpassaged in Balb/c Rag-2^-/-^/Jak3^-/-^ mice. A shorter duration of PDX growth was observed during F0 to F2 transplantation; concomitantly, increased Oct-3/4 and Sox2 were evidenced in 50% and 33%, respectively, of serial PDXs. Only four cell lines were established. The cell lines exhibited either bile duct (KKK-D049 and KKK-D068) or combined hepatobiliary origin (KKK-D131 and KKK-D138). These cell lines acquired high transplantation efficiency in both subcutaneous (100%) and intrasplenic (88%) transplantation models. The subcutaneously transplanted xenograft retained the histological architecture as in the patient tissues. Our models of CCA PDX and PDX-derived cell lines would be a useful platform for CCA precision medicine.

## 1. Introduction

Cholangiocarcinoma (CCA) is a rare subtype of liver cancer for which the highest incidence and mortality have been reported in northeastern Thailand [1,2]. The prognosis of CCA is dismal because of delayed diagnosis and poor response to conventional chemotherapy and targeted treatment [3]. Surgery is the only treatment option that provides a curative outcome [3,4], but limited numbers of the patients are candidates [5]. Nonetheless, this outcome is influenced by factors such as tumor subtype, complete resection (R0), lymph node involvement, and vascular invasion [6]. Moreover, more than 85% of patients suffer from the disease recurrence [6]. The benefits of postoperative adjuvant treatment from a recent systematic review and meta-analysis are not convincing [7]. Therefore, it is urgently important to develop a novel CCA treatment.

CCA has high heterogeneity in nature [8]. Several risk factors of CCA have been established [9]. A common risk factor for of CCA in Thailand is the presence of a liver fluke, *Opisthorchis viverrini* (*Ov*), through ingestion [1,10]. A unique feature of *Ov*-associated CCA is increased xenobiotic metabolizing gene expression [11]. Mutational analysis of *Ov*-associated CCA identified a distinct signature with higher rates of *TP53*, *SMAD4*, and *ARID1A* mutations but lower frequencies of *IDH1/2* and *BAP1* mutations when compared to other Asian and Western populations [12,13,14]. These differing characteristics might complicate the usage of newly identified targets for CCA treatment [15,16,17]. However, the recent advances in multi-omics analyses have led to the identification of the targets for Thai CCA [18,19]. The majority of *Ov*-associated CCAs were gathered in cluster 1 and cluster 2 in Jusakul’s study [18]. The molecular signature of cluster 1 showed some degree of similarity to CCA-C1 subgroup in Chaisaingmongkol’s study [19] and to the proliferation subclass in Sia’s study [17]. Potential usage of Her2/neu (ERBB2) [18] and cycle regulatory molecules [19] as targets for treatment are suggested in these CCA subclasses. Therefore, the development of a model for CCA treatment prediction and validation is urgent.

There are several models for CCA, including a previously established cell line, a cell line-transplanted xenograft, and a genetically engineered mouse model. The limitation of these models is disease homogeneity [20,21]. The generation of a patient-derived model might be better representative of tumor biology. However, primary culture of patient tissue is laborious and less efficient. In highly desmoplastic tumors (e.g., CCA and pancreatic cancer), the overgrowth of stromal cells will reduce the establishment efficiency [22,23]. In our experience, the success rate of cell line establishment from patient-derived primary culture was less than 5%, (unpublished data), comparable to that of pancreatic cancer (7%) [22].

The patient-derived xenograft (PDX) model is a promising tool for the propagation of a patient’s tumor in an immunodeficient mouse. This PDX is an invaluable asset for the advancement of cancer precision medicine, particularly for rare and aggressive cancers [24]. A higher success rate of cell line development from PDX has been reported [25,26,27]. These cell lines show a certain degree of disease heterogeneity [26]. Limited numbers of CCA PDX and PDX-derived cell lines are available [16,27,28]. This prompted us to develop PDX as well as the PDX-derived cell line for high-throughput drug screening. These models might be useful as a platform for future anti-CCA development.

## 2. Materials and Methods

### 2.1. Cell Line

Four CCA cell lines—KKU-055, KKU-100 [29], KKU-213, and the hepatocellular carcinoma (HCC) cell line-HuH-7 [30] were selected as reference liver cancer cell lines for current study. KKU-055 and KKU-213 were derived from intrahepatic CCA, while KKU-100 was derived from the extrahepatic (perihilar) CCA [29]. CCA cell lines were obtained from the Japanese Collection of Research Bioresources Cell Bank (Osaka, Japan). HuH-7 was kindly provided by Prof. Kyoko Tsukiyama-Kohara (Kagoshima University, Kagoshima, Japan). Cells were maintained in DMEM (Wako, Osaka, Japan) or RPMI1640 (Wako) as per recommendations. FBS (10%; HyClone, Logan, UT, USA), 100 U/mL penicillin, and 100 μg/mL streptomycin were supplemented in the media. Cultures were maintained at 37 °C in a humidified 5% CO_2_ atmosphere.

### 2.2. CCA Tissue Collection and Storage

Sixteen CCA tissue samples were obtained from the Department of Pathology, Faculty of Medicine, Khon Kaen University, Thailand, after the clinical specimens were obtained from the operating room and the pathological specimens were taken as the standard protocol for pathological staging. All tissue samples were histologically diagnosed; 10 were of intrahepatic CCA (ICC), and six were of the extrahepatic subtype (ECC). The study protocol was reviewed and approved by the Ethical Committee for the Human Research of Khon Kaen University (HE571283), based on the Declaration of Helsinki of 1975. Written informed consent was obtained from each subject. Demographical and pathological characteristics of patients and CCA tissues are listed in Table 1. CCA tissues were cut into 0.5 × 0.5 × 0.5 cm pieces and were stored in the freezing media containing 10% DMSO and 90% FBS, with 3–4 pieces per frozen vial. Tissues were stored at −80 °C until needed.

For transplantation, frozen CCA tissues were thawed and vigorously washed with PBS three times. Tissues were cut into 8–27 mm^3^ pieces (2–3 mm each dimension). Non-viable cells were removed. Each tissue was divided into two parts: (1) for transplantation and (2) for molecular characterization and paraffin-embedded tissue preparation. Tissues were transplanted into flank areas of Balb/c Rag-2/Jak3 double-deficient (Balb/c RJ) [31] or Balb/c nude Rag-2/Jak3-deficient (Nude RJ) mice [32] subcutaneously. Implanted tissues were observed three times a week and were removed when the masses reached 8–10 mm in diameter. Xenograft tumors from mice were sub-divided into four parts: (1) for serial transplantation, (2) for frozen tissue stock, (3) for cell line development, and (4) for histological purposes. All experimental protocols were approved by The Institutional Animal Care and Use Committee, Kumamoto University, Japan.

### 2.3. Cell Line Establishment

For cell line development, fresh xenograft tissues were prepared as previously described [34]. Cells were cultured in DMEM/F12 (Wako) containing 1–10% FBS and insulin-transferrin-selenium (ITS, Gibco BRL, Carlsbad, CA, USA). Stromal cells were sequentially removed by partial trypsinization and mechanical removal. Cancer cells were subsequently cultured in DMEM containing 10% FBS when becoming morphologically homogenous. All media were supplemented with 100 U/mL penicillin and 100 μg/mL streptomycin. The cultures were maintained at 37 °C in a humidified 5% CO_2_ atmosphere. Cells were maintained in vitro culture system at least 6 months to ensure the immortalization properties.

All four newly established cell lines were deposited into the Japanese Cancer Research Resources Bank (JCRB), National Institutes of Biomedical Innovation, Health and Nutrition (NIBIOHN), Osaka, Japan.

### 2.4. The Expressions of Bile Duct and Hepatocyte-Related Genes

To demonstrate the liver cell origin of the cell lines, the expression profile of bile duct or hepatocyte-related genes including cytokeratin 7 (CK7), CK19, γ-glutamyl transferase (GGT), α-fetoprotein (AFP), and albumin (ALB) were determined as previously described [35]. RNA was isolated from the cell line, and cDNA was prepared as mentioned elsewhere [36]. Alpha-smooth muscle actin (αSMA) primers were used for the exclusion of fibroblast contamination. Primers used in the current experiment are listed in Table S1.

PCR products were separated in 1.5% agarose gel in Tris-Borate-EDTA (TBE) buffer. A gel was stained with ethidium bromide solution (Sigma-Aldrich, St. Louis, MO, USA) and the images were captured by Bio-Rad Gel Doc 2000 (Bio-Rad, Hercules, CA, USA).

### 2.5. Cell Line Authentication and TP53 Mutation Analysis

To determine the genetic stabilities of the cell lines, 16 short tandem repeats (STR) of cell lines, original tumor tissues, and patient’s white blood cells (WBC) were compared using AmpFℓSTR^®^ Identifiler^®^ Plus PCR Amplification Kit (Applied Biosystems, Carlsbad, CA, USA). DNA was extracted by the QIAamp^®^ DNA Micro Kit (QIAGEN, Stanford, CA, USA). PCR products were analyzed using ABI Prism 3130 Genetic Analyzer and GeneMapper^®^ ID Software v3.2 (Applied Biosystems).

*TP53* gene mutation was analyzed as previously described in [37]. Briefly, PCR reactions were performed using the HotStarTaq Master Mix Kit (QIAGEN) and the amplification reactions were carried out on a GeneAmp 9700 Thermal cycler (Applied Biosystems) as suggested. Sequencing was achieved by using BigDye Terminator V3.1 cycle sequencing reaction kit (Applied Biosystems) and the Genetic Analyzer ABI 3130 (Applied Biosystems). TP53 sequences were compared to the reference sequence (NC_000017.9) by Lasergene 10.1 (DNASTAR, Madison, WI, USA).

### 2.6. Xenograft Transplantation of Cell Lines

The xenograft transplantation ability of the cell lines was determined. One to two million cells of each cell line were transplanted subcutaneously into both flanks of Balb/c RJ mice. For intrasplenic transplantation, 5 × 10^4^ cells were injected intrasplenically as previously described [38]. Tissues, spleen, and liver were removed at 1 month after transplantation. Paraffin-embedded tissues were prepared as per standard protocol.

### 2.7. Histological Characterization and Evaluation

Hematoxylin and eosin staining of the original CCA tissues and transplanted tumors was performed regularly. For immunohistochemistry staining, a standard protocol using citrate buffer retrieval buffer was used. Signals were enhanced by EnVision-system-HRP (Dako, Glostrup, Denmark) or the Vectastain Elite ABC standard kit (Vector Laboratories, Burlingame, CA, USA). Detection was performed using the Histofine^®^ DAB substrate kit (Nichirei Bioscience, Tokyo, Japan).

The sources of antibodies were as follows: anti-CK19 (HPA002465,) was from Sigma-Aldrich, anti-Ki-67 (MIB-1) was from Dako, anti-epithelial cell adhesion molecule (EpCAM, C-10) and anti-Oct-3/4 (C-10) were from Santa Cruz Biotechnology (Dallas, TX, USA), anti-Sox2 (D6D9) was from Cell Signaling Technology (Danvers, MA, USA), and biotinylated goat anti-mouse IgG and biotinylated goat anti-rabbit IgG were from Vector Laboratories.

The comparison of tissue architecture between the original CCA tissue and transplanted tissue was made by the pathologists. The images were taken by the BZ-8100 Biozero fluorescent microscope. For the quantitation, the immunoreactivity signals were quantified by BZ-II Analyzer (Keyence, Osaka, Japan) as previously described [36].

### 2.8. Statistical Analysis

For the correlation study, Pearson’s correlation coefficient (*r*) was calculated using GraphPad Prism version 6.07 (San Diego, CA, USA).

## 3. Results

### 3.1. CCA Patient Tissue Transplantation

**Figure 1:**
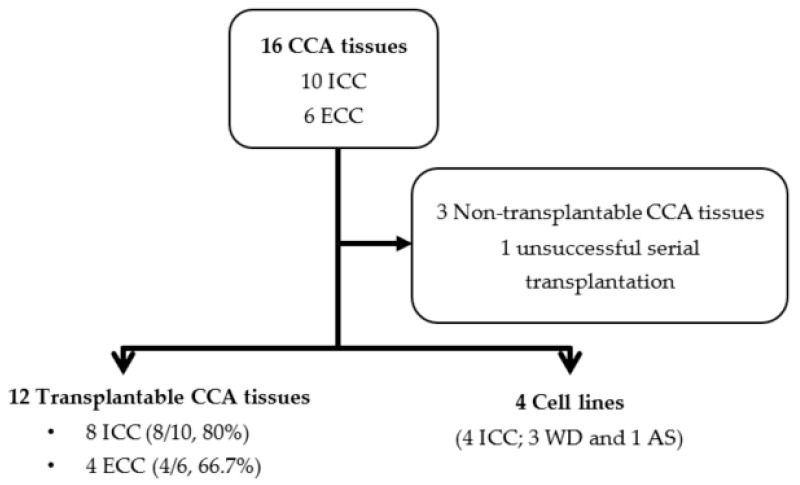
The summary of PDX transplantation and PDX-derived cell line development. CCA: cholangiocarcinoma.

From 16 CCA tissues, 10 tissue samples were of the intrahepatic subtype (ICC) while six were of the extrahepatic subtype (ECC) based on the 7th edition of the AJCC cancer staging classification. The most extended storage duration with successful transplantation was 134 days (19–134 days). After defrosting and cleaning, tissues were transplanted into both flanks of Balb/c RJ mice. Thirteen tissue samples (eight ICC and five ECC) successfully grew in the subcutaneous areas of the mice (Table 1). No mass was observed in three mice (D039, D042, and D117). Unfortunately, only 12 tissue samples were successfully transplanted into F1 (Figure 1). D106 formed a tumor in the F0 mouse, but it was lethal to the F1 mouse. We repeated D106 F1 transplantation twice but mice died within a month without a specific cause and alarming signs. The durations of F0 tumor formations ranged from 24 to 194 days. The duration of tumor formation was not related to either tumor cell density evaluated by percentage of CK19 immunoreactivity or the proliferative potential of the tumor cells determined by percentage of Ki-67 positive nuclei (Table S2 and Figure S1).

A similar experiment was carried out in nude RJ mice. Three samples were transplanted into both flanks of mice. The transplantation success rate was comparable to those in Balb/c RJ, but F0 growth in nude RJ took approximately 2 weeks longer. Thus, we selected the Balb/c RJ mice for our PDX generation and further testing.

**Figure 2:**
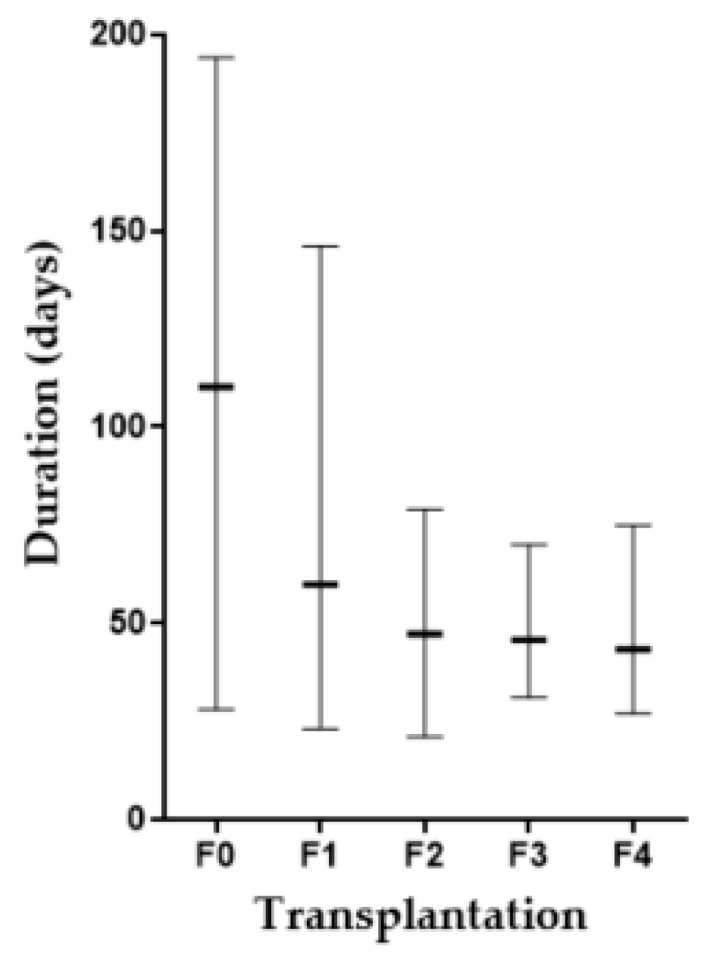
Comparison of PDX growth duration during serial transplantation. Bar indicates the longest to the shortest duration in each generation and - indicates a mean duration.

**Figure 3:**
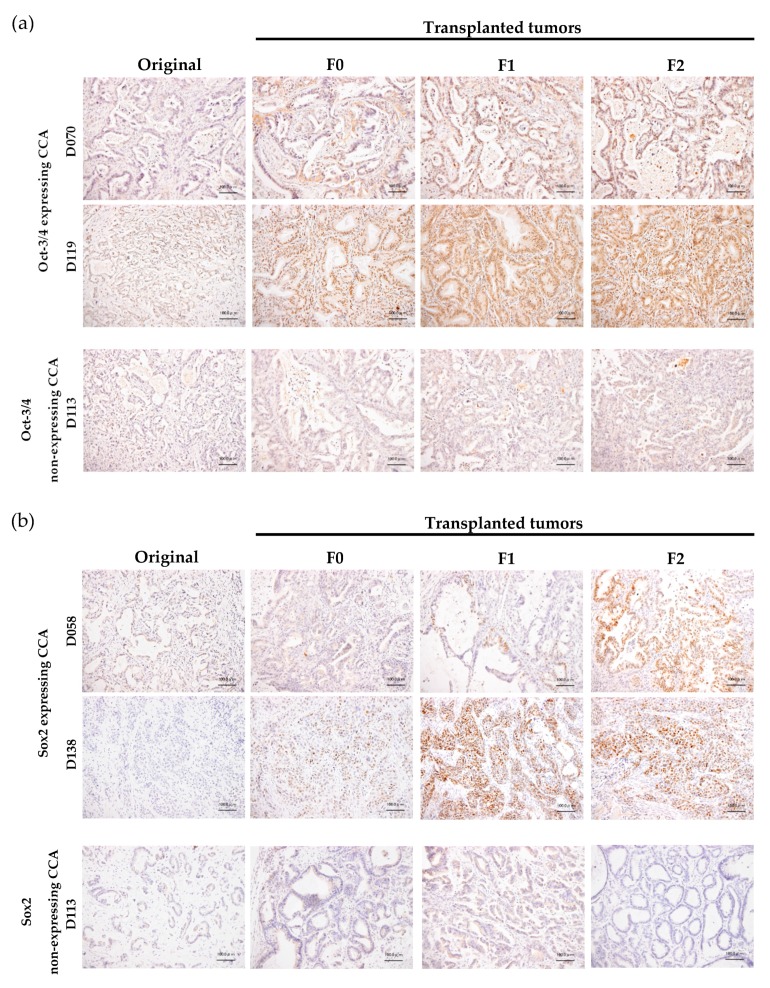
Comparison of Oct-3/4 and Sox2 expressions between original tumor tissue from the patient (original) and serially transplanted tissues (F0, F1, and F2). (**a**) Oct-3/4; (**b**) Sox2 expressions. Representative samples of nuclear expressing and non-expressing CCA are shown. Bar = 100 μm.

From our protocol, we successfully established the method for generation of the CCA-PDX model, which is highly efficient (67–80% success rate) (Figure 1). The duration of xenograft transplantation was not related to either tumor density or the proliferative capability of the tumor cells indicated by CK19 and Ki-67 immunostaining. Nonetheless, it is worth mentioning that the duration of tumor establishment was shorter when the PDX was serially transplanted (Figure 2). Mean durations of PDX growth in F0, F1, F2, F3, and F4 were 110, 60, 47, 46, and 43 days, respectively. The xenograft tumor growth might be related to the Oct3/4 and Sox2 expressions. Oct-3/4 was detectable in 11 xenografts (92%) and Sox2 was observed in eight xenografts (67%). Interestingly, increased Oct3/4 was observed in 50% of serially transplanted tumors (6/12 xenografts) and increased Sox2 was observed in 33% of tumors (4/12 xenografts). Representative PDXs with Oct-3/4 and Sox2 increments are demonstrated in Figure 3. It should be noted that EpCAM was observed on almost all tumor cell surfaces and no significant alteration of the EpCAM signal was observed in our serially transplanted PDXs (Figure S2).

### 3.2. Cell Line Establishment and Characterization

**Figure 4:**
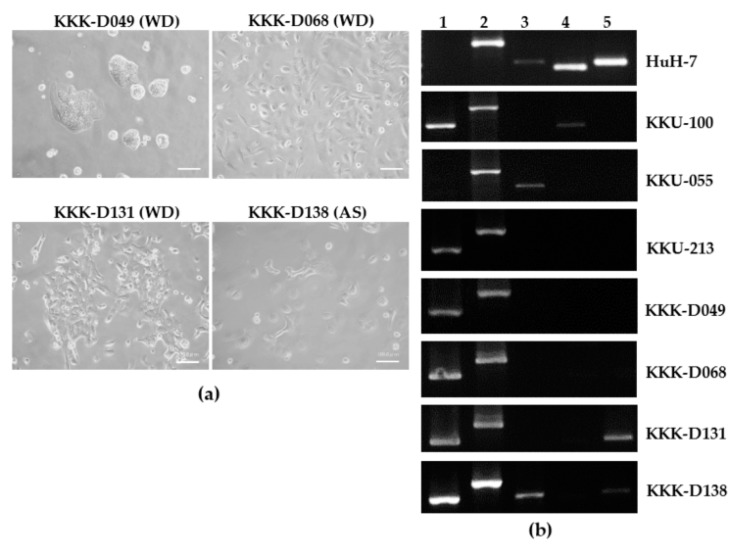
The morphologies (**a**) and gene expression profile (**b**) of PDX-derived CCA cell lines KKK-D-49, KKK-D068, KKK-D131, and KKK-D138. Huh7, KKU-100, KKU-055, and KKU-213 were used as references. WD: well differentiated subtype; AS: adenosquamous subtype; 1: cytokeratin 7 (CK7); 2: CK19, 3: γ-glutamyl transferase (GGT); 4: α-fetoprotein (AFP); 5: albumin (ALB).

Among 12 serially transplantable tissues, four tissues were successfully developed into cell lines. All cell lines were of intrahepatic origin; three were histologically characterized as well-differentiated subtypes (WD; KKK-D049, KKK-D068, and KKK-D131) and one was characterized as a mixed adenosquamous subtype (AS; KKK-D138). KKK-D068 and KKK-D131 were established from the F0-transplanted tumor while KKK-D138 and KKK-D049 were established from the F1 and F2 tumors, respectively. The morphologies of the cell lines are presented in Figure 4a. All cell lines exhibit epithelial-like features with a high nuclear to cytoplasmic ratio. KKK-D049 shows a unique feature of tight clustering. KKK-D068, KKK-D131, and KKK-D138 contain both polygonal and spindle-like cells.

The authentications of cell lines were performed by STR analysis (Table 2). DNA from patient’s tissue and WBC were used as references. Fifteen STR loci and amelogenin were detected. One locus of D18S51 was lost in the D049 tissue and KKK-D049 cell line. One locus of D16S539 and Y amelogenin was lost in the KKK-D068 cell line but not in D068 tissue. A locus of CSF1PO, TH01, D16S539, D19S433, D5S818, FGA, and Y amelogenin was lost in KKK-D131. A 31.2 locus of D21S11 was lost in KKK-D138.

Further characterization of cell lineage marker was performed using RT-PCR (Figure 4b). Comparisons of the previously developed HCC (HuH-7), CCA (KKU-100, KKU-055, KKU-213) and the newly established cells showed all new cell lines expressed two bile duct markers, CK7, and CK9, similar to the previously established cell lines (KKU-100 and KKU-213); only KKK-D138 expressed GGT. KKK-D131 and KKK-D138 expressed the hepatocyte marker, ALB. None of the newly established cell lines expressed AFP but a previously developed cell line, KKU-100 did. Alpha-SMA was not detectable in any cell lines (data not shown).

*TP53* mutation analysis revealed CCC to CGC at codon 72, which will cause missense P72R mutations in KKK-D138 but is not detected in KKK-D068 and KKK-D131. The *TP53* mutation of KKK-D049 has not yet been analyzed.

### 3.3. Cell Line Transplantation

**Figure 5:**
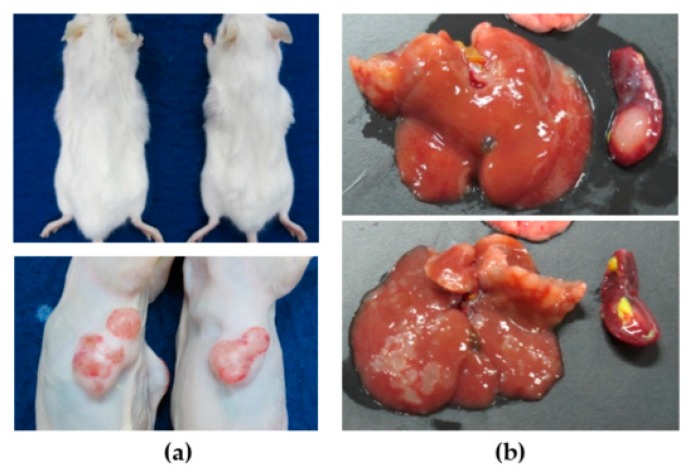
CCA cell line transplantation in subcutaneous (**a**) and intrasplenic (**b**) xenograft mouse models. The representative pictures are from KKK-D068 transplantations.

To test the in vivo tumorigenesis properties of the newly developed cell lines, the cell lines were separately injected into both flanks of Balb/c RJ (two mice/cell line, *n* = 4). Tumors were grown in the mice for a month, and tumor masses were observed twice a week. We observed tumor masses from all injected sites (Figure 5a). The successful rate of subcutaneous xenograft was 100% (Table 3).

The transplantation was further performed in the intrasplenic transplantation model. Fifty thousand cells of each cell line were injected into Balb/c RJ spleen (two mice/cell line, *n* = 2). Spleen, liver, and lungs were removed at 1 month after injection. Tumors were observed in 88% of transplanted livers and spleens (7/8 mice) (Figure 5b). No tumors were detected in one mouse injected with KKK-D049 (Table 3). No tumors were observed in lungs of mice (data not shown).

**Figure 6:**
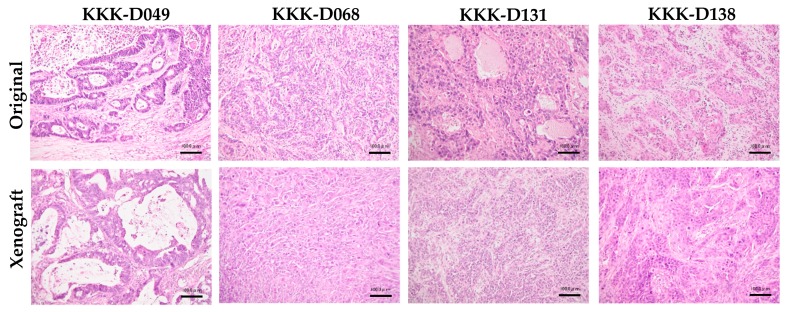
Histological comparison of original tumor tissues from patient (original) and subcutaneous transplanted tissues (xenograft). Bar = 100 μm.

The subcutaneous tumor masses were prepared for the histological comparison with the tumor tissues from the patients (Figure 6). These transplanted tumors exhibited similar architectures to the original tumors. KKK-D049 showed tubular formation, while KKK-D068, KKK-D131, and KKK-D138 exhibited epithelial like characteristics.

## 4. Discussion

Cholangiocarcinoma (CCA) is a rare, aggressive tumor of liver found worldwide [1,2]. The world’s highest incidence and mortality of CCA are in Thailand, and the disease is a significant health concern. CCA treatment is difficult given the great heterogeneity of the disease. The unique causative agent of *Opisthorchis viverrini* (*Ov*) infection has been demonstrated by distinctive molecular signatures [11,12,13,18,19]. The typical signatures suggest *Ov*-related CCA might be vulnerable to distinct treatment. Several attempts were made to provide opportunities for *Ov*-related CCA treatment [18,19]. Despite this, pre-clinical models that are patient-representative are limited. Therefore, a model for target validation is urgently required.

Patient-derived xenograft (PDX) models are preclinical models that are ideally developed for the implementation of personalized medicine. PDX models are a powerful tool for cancer propagation that retain disease complexity and heterogeneity [24]. Moreover, PDX-derived cell lines with some degree of cellular heterogeneity are useful tools for the larger scale of drug screening. We have developed the PDX and PDX-derived cell lines which acquire very high efficiency for transplantation in conventional subcutaneous and intrasplenic models.

The newly developed CCA-PDX model is highly efficient, with 75% transplantable efficiency compared to 6–35% engraftment rates in the previously described models [27,28]. This high efficiency is not related to the tumor stage, patient survival, tumor density, or proliferative potential of cells. This might be due to the different genetic backgrounds of the recipient mice. Balb/c RJ or nude RJ mice in our study have no natural killer (NK) cells, but the non-obese diabetic (NOD)/Shi-severe combined immunodeficient (SCID) mice in Cavalloni’s study and the athymic C.B17/Icr-scid(scid/scid) mice in Ojima’s study retain functional NK cells [27,28]. The roles of NK cells in syngeneic or xenograft tumor rejection are widely accepted [39]. The usefulness of the PDX model has been explored as the resources for cell line development [25,26,27]. A comparable cell line establishment rate (25% in our study vs. 32% in Ojima’s study) was observed, which might be due to the selective power of the PDX model [20]. Increased Oct-3/4 or Sox2 expressing cancer cells were observed during PDX passaging. Similar observations of increased cancer stem cell (CSC) proportions in the PDX model were observed in other cancers [40]. The identification and characterization of CSCs in CCA-PDX are beyond the scope of this study and require more attention.

PDX-derived cell lines developed in this study show some degree of heterogeneity in vitro and in vivo, yet keep the characteristics of tissue organization, which are common in PDX models [25,26,27]. The loss of the Y chromosome or Y amelogenin was observed in two cell lines (KKK-D068 and KKK-D131). The loss of the Y chromosome is also observed in hepatocellular carcinoma (HCC) [41] and pancreatic cancer [42]. The functional significance of the Y chromosome loss is still under debate. Our PDX-derived cell lines acquire the expressions of the bile duct or hepatobiliary-related genes. This might suggest a cellular origin, perhaps committed bile duct cells (CK7- and CK19-expressing cells) or bipotential progenitor cells (CK7/CK19 and ALB-expressing cells) [35]. Cellular origins of cell lines require further investigation. The newly established cell lines have very high efficiency for xenotransplantation. Owing the PDX development was in Balb R/J, these cell lines might be adapted to the selection power of the mouse model. Testing of xenotransplantation efficiency in other immunodeficient will be explored.

**Figure 7:**
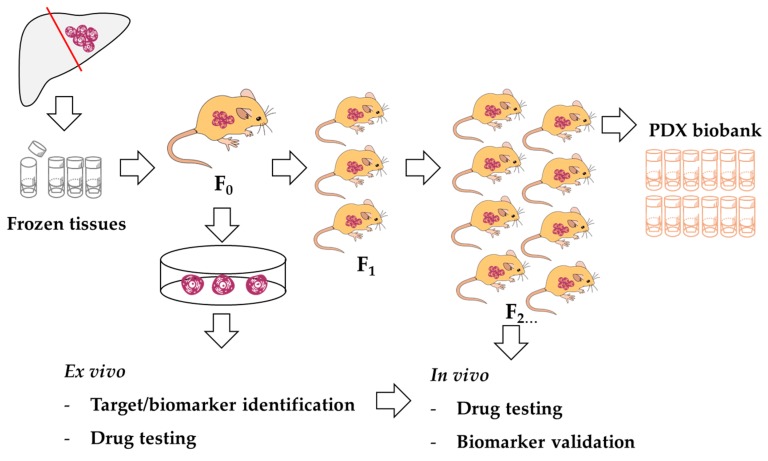
A proposed model of PDX resource usage.

We propose the platform for anti-cancer drug screening using a PDX-derived cell line, and a PDX mouse model as demonstrated in Figure 7. In cases where immunodeficient mice are not commonly available, cancer tissues might be kept as frozen stock. Transplantation might be performed upon readiness. In parallel, the omics identification of drug targets and the development of PDX-derived cell lines might be performed. This cell line might be useful for high-throughput drug screening or target testing. To validate the information from omics study or high-throughput drug screening, the PDX model may play a critical role. Moreover, this PDX biobank would be a precious resource for future drug development.

## 5. Conclusions

In conclusion, in this study we established a highly effective CCA PDX model and highly transplantable PDX-derived cell lines. Cell authentications and characterization have been demonstrated. The cellular heterogeneity and preserved tissue architecture have been confirmed in PDX-derived cell lines and cell line xenografts. These PDX-derived cell lines and PDX models might be a promising platform for anti-CCA development. The advantage of our PDX model for personalized medicine seems to be limited by the long transplantation duration in some cases.

## Figures and Tables

**Table 1:** Characteristics of patients and cholangiocarcinoma tissues.

Code	Gender	Age	Subtype	TMN **	Stage **	*Ov* ^#^	PDX ^##^	Histological Classification
D039	F	66	ICC	T3N0M0	III	No	−	WD, papillo-tubular adenocarcinoma
D042	M	56	ECC	T2bN0M0	II	No	−	Invasive, intraductal papillary carcinoma
D049 *	M	55	ICC	T2bN0M0	II	*Ov*	+	WD, tubular adenocarcinoma
D058	F	64	ICC	T3N1M0	IVA	*Ov*	+	WD, tubular adenocarcinoma
D068 *	M	61	ICC	T2aN1M0	IVA	No	+	WD, tubular adenocarcinoma with micropapillary foci
D070	M	65	ICC	T3N1M0	IVA	No	+	WD, tubular adenocarcinoma
D078	F	44	ECC	T4N1M0	IVA	No	+	WD, tubular adenocarcinoma
D088	F	68	ICC	T3N0M0	III	No	+	MD, tubular adenocarcinoma
D090	F	65	ECC	T2bN0M0	II	No	+	Invasive, intraductal papillary carcinoma
D096	M	45	ECC	T3N1M0	IIIB	No	+	WD, tubular adenocarcinoma
D106	M	54	ECC	T2bN1M0	IIIB	No	+/−	Invasive, intraductal papillary carcinoma
D113	M	70	ICC	T3N0M0	III	No	+	Invasive, intraductal papillary carcinoma
D117	M	58	ICC	T3N1M0	IVA	No	−	WD, tubular adenocarcinoma with micropapillary foci
D119	M	71	ECC	T3N1M0	IIIB	No	+	WD, tubular adenocarcinoma
D131 *	M	66	ICC	T3N1M0	IVA	No	+	WD, tubular adenocarcinoma
D138 *	F	60	ICC	T3N0M0	III	No	+	Adenosquamous carcinoma

* Cell lines were established; ** classification is based on the 7th edition of the AJCC cancer staging classification [33]; ^#^ *Opisthorchis viverrini* (*Ov*) is observed in the tissues; ^##^ serial transplanted tissues are successfully established, +/− indicates only F0 tumor was obtained; F: female; M: male; ECC: extrahepatic cholangiocarcinoma; ICC: intrahepatic cholangiocarcinoma; MD: moderately-differentiated subtype; WD: well-differentiated subtype; PDX: patient-derived xenograft.

**Table 2:** Comparison of STR profiles of CCA tissues, patient WBC, and newly established cell lines.

Loci	D049			D068			D131			D138		
	WBC	Tissue	Cell	WBC	Tissue	Cell	WBC	Tissue	Cell	WBC	Tissue	Cell
D8S1179	12, 17	12, 17	12, 17	12, 16	12, 16	12, 16	12, 13	12, 13	12, 13	ND **	10, 14	10, 14
D21S11	29, 30	29, 30	29, 30	30, 33.2	30, 33.2	30, 33.2	29	29	29		29, 31.2	29
D7S820 *	8, 10	8, 10	8, 10	8, 10	8, 10	8, 10	8, 11	8, 11	8, 11		10, 11	10, 11
CSF1PO *	11, 12	11, 12	11, 12	11	11	11	12, 13	12, 13, 14	14		10, 11	10, 11
D3S1358	15, 16	15, 16	15, 16	15	15	15	14, 15	14, 15	14, 15		16, 18	16, 18
TH01 *	9	9	9	7	7	7	8, 9.3	8, 9.3	9.3		8, 9.3	8, 9.3
D13S317 *	8, 9	8, 9	8, 9	8, 12	8, 12	8, 12	10, 11	10, 11	10, 11		8, 11	11
D16S539 *	13, 14	13, 14	13, 14	9, 11	9, 11	9	9, 11	9, 11	11		9, 11	9, 11
D2S1338	19, 25	19, 25	19, 25	19	19	19	20, 23	20, 23	20, 23		24, 25	24, 25
D19S433	13, 15.2	13, 15.2	13, 15.2	14, 14.2	14, 14.2	14, 14.2	13.2, 14	13.2, 14	13.2		13.2, 14.2	13.2, 14.2
vWA *	14, 17	14, 17	14, 17	14, 16	14, 16	14, 16	14, 16	14, 16	14, 16		14, 18	14, 18
TPOX *	8, 9	8, 9	8, 9	8, 11	8, 11	8, 11	8, 11	8, 11	8, 11		11	11
D18S51	11, 16	11	11	12	12	12	17	17	17		15	15
D5S818 *	10, 12	10, 12	10, 12	11, 12	11, 12	11, 12	10, 12	10, 12, 13	13		9, 10	9, 10
FGA	23, 24.2	23, 24.2	23, 24.2	23, 25	23, 25	23, 25	19, 21	19, 21	21		18, 24.2	18, 24.2
Amelogenin	X, Y	X, Y	X, Y	X, Y	X, Y	X	X, Y	X, Y	X		X, X	X, X

* Eight markers are common short tandem repeat (STR) markers for cell authentication; ** White blood cells (WBCs) are not available for comparison.

**Table 3:** PDX-derived cell line transplantation rate in subcutaneous (SC) and intrasplenic (IS) xenograft mouse model.

Cell Lines	Route	Transplantation Rate (%)
KKK-D049	SC	4/4 (100%)
	IS	1/2 (50%)
KKK-D068	SC	4/4 (100%)
	IS	2/2 (100%)
KKK-D131	SC	4/4 (100%)
	IS	2/2 (100%)
KKK-D138	SC	4/4 (100%)
	IS	2/2 (100%)

## Supplementary Materials

Supplementary materials are available online at https://www.mdpi.com/2073-4409/8/5/496/s1.
