# Supplementary material for: Establishment of Highly Transplantable Cholangiocarcinoma Cell Lines from a Patient-Derived Xenograft Mouse Model
Source: Cells. 2019 May 23;8(5):496. doi: 10.3390/cells8050496 (PMC6562875; doi:10.3390/cells8050496)
Supplement: Supplementary file 1 [file cells-08-00496-s001.pdf]

cells MDPI

Article

Establishment of highly Transplantable Cholangiocarcinoma Cell Lines from Patient Derived Xenograft Mouse Model

Table 1. Primers for CCA cell line characterization.

Genes	Primer sequence (5'→3')		Products
	Sense	Antisense	(bp)
AFP	TTTTGGGACCCGAACTTTCC	CTCCTGGTATCCTTTAGCAACTCT	451
ALB	GGTGTTGATTGCCTTTGCTC	CCCTTCATCCCGAAGTTCAT	502
CK7	CCCTCAATGAGACGGAGTTG	GTAGGTGGCGATCTCGATGT	497
CK19	TCCCGCGACTACAGCCACTAC TACACGACC	CGCGACTTGATGTCCATGAGCC GCTGGTAC	746
GGT	ACAACAGCACCACACGAAAA	TCAGCTCAGCACGGTAGTTG	501
αSMA	CTTCAGCTTTCAGCTTCCCTGA	TTGCTCTGTGCTTCGTCACC	274

Table 2. Durations of the F0 tumor developments, patient survival times, percent of CK19 positive area and percent of Ki-67 positive nuclei.

Code	Survival* (days)	Duration** (days)	%CK19	%Ki-67
D049	257	58	27	40
D058	105	123	17	30
D068	212	28	37	5
D070	208	144	46	10
D078	193	163	2	10
D088	165	106	39	5
D090	161	163	39	0.1
D096	144	39	19	0.1
D113	94	152	25	0
D119	71	194	15	5
D131	31	47	63	10
D138	23	111	32	5

*Survival was calculated from the surgery to death; **Duration was calculated from implantation to removal.

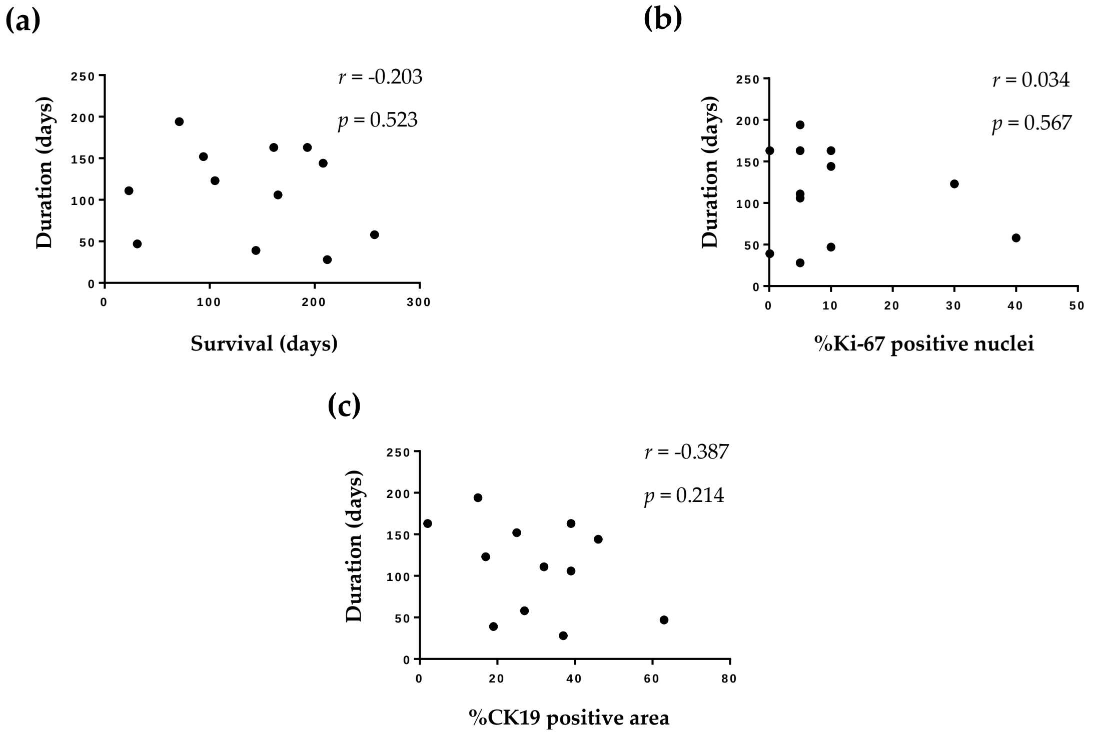

Figure 1. The correlation of the F0 tumor development and patient survival, percentage of Ki-67 positive nuclei and percentage of CK19 positive area.

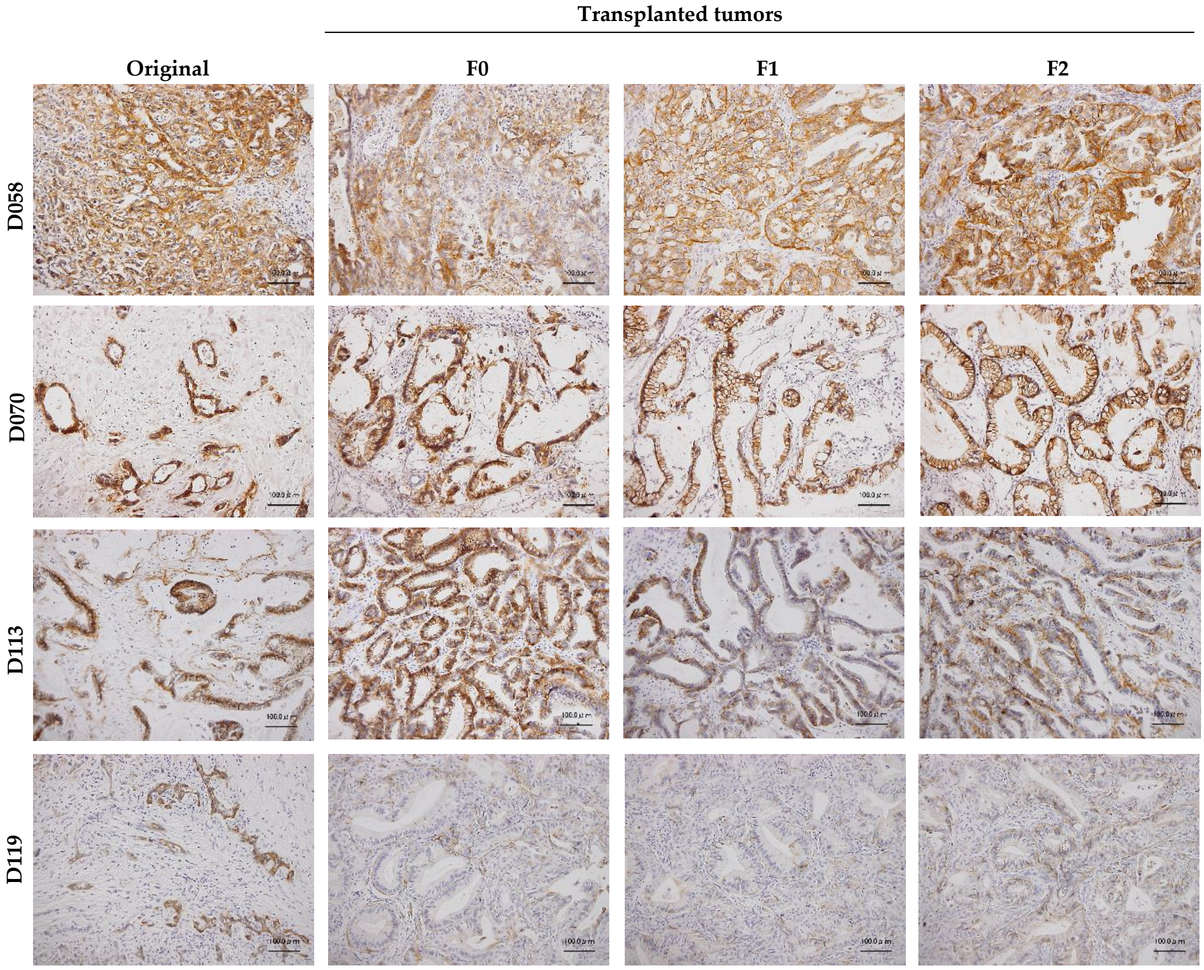

Figure 2. Comparison of EpCAM expressions between original tumor tissues from patient (original) and serially transplanted tissues (F0, F1 and F2). Bar = 100 µm.
